# Production of Protocatechuic Acid in *Bacillus Thuringiensis* ATCC33679

**DOI:** 10.3390/ijms13033765

**Published:** 2012-03-21

**Authors:** Kimtrele M. Williams, William E. Martin, Justin Smith, Baraka S. Williams, Bianca L. Garner

**Affiliations:** 1 Department of Biology, Tougaloo College, Tougaloo, MS 39174, USA; E-Mails: kwilliams@yahoo.com (K.M.W.); wmartin601@live.com (W.E.M.); smithsmtty2@aol.com (J.S.); williamsbaraka@yahoo.com (B.S.W.); 2 Center for Bioinformatics and Computational Biology, Department of Biology, Jackson State University, Jackson, MS 39217, USA

**Keywords:** catechol, *Bacillus*, protocatechuic acid

## Abstract

Protocatechuic acid, or 3,4-dihydroxybenzoic acid, is produced by both soil and marine bacteria in the free form and as the iron binding component of the siderophore petrobactin. The soil bacterium, *Bacillus thuringiensis kurstaki* ATCC 33679, contains the *asb* operon, but does not produce petrobactin. Iron restriction resulted in diminished *B. thuringiensis kurstaki* ATCC 33679 growth and the production of catechol(s). The gene product responsible for protocatechuic acid (*asbF*) and its receptor (*fatB*) were expressed during stationary phase growth. Gene expression varied with growth temperature, with optimum levels occurring well below the *Bacillus anthracis* virulence temperature of 37 °C. Regulation of protocatechuic acid suggests a possible role for this compound during soil growth cycles.

## 1. Introduction

Iron uptake mechanisms in members of the *Bacillus cereus sensu lato* group have emerged as areas of interest in the identification of virulence factors and as diagnostic tools. Iron is required by almost all living organisms because of the central role it plays in biochemical reactions. Although iron is an abundant metal, in an aerobic environment it binds oxygen producing insoluble ferric hydroxide particulates, making it difficult to access for normal biological functions. Most microorganisms secrete siderophores, iron chelators, to remove and sequester iron in diverse environments. Catecholates are the most common type of siderophores produced and are classically described as containing the simple catechol 2,3-dihydroxybenzoic acid (2,3-dhb) [1]. *B. cereus* and *Bacillus anthracis* produce a 2,3-dhb containing siderophore, bacillibactin, and a 3,4-dihydroxybenzoic acid (3,4-dhb) containing siderophore, petrobactin [2]. The hydroxyl groups of the catechol rings form the iron chelation center, resulting in a hexadentate interaction with iron, creating the siderophores high affinity for iron [1].

Production of both the simple catechols and the siderophores are regulated by the environment. Most importantly, growth temperature differentially regulates production in *B. anthracis*, with greater quantities of petrobactin produced at the virulence temperature of 37 °C [3]. While both siderophores are regulated by iron availability, some *B. anthracis* genes associated with petrobactin production are less sensitive to iron concentration [4]. Indeed, there is no annotated ferric uptake regulator box, an iron-responsive regulator sequence, within the petrobactin biosynthetic pathway [5]. Regardless of the growth conditions, *B. anthracis* produces large amounts of 3,4-dhb, or protocatechuic acid, which has not been demonstrated to reverse iron restricted growth [6] while 2,3-dhb has been demonstrated to act as a siderophore for some microbes [7]. It is also unclear how the gene specifically responsible for 3,4-dhb production, *asbF*, is regulated under these conditions, as it was not measured in the previous *B. anthracis* studies.

There is limited information concerning siderophore regulation in *Bacillus thuringiensis*, a routine environmental contaminant [8]. *B. thuringiensis* is an ubiquitous soil bacteria and is a common component of organic pesticides. A member of the *B. cereus* group, this large Gram-positive microbe secretes paracrystalline toxins that kill numerous insects. *B. thuringiensis* and *B. cereus* are routinely used as non-pathogenic models for *B. anthracis* because of the genetic similarity and the mechanisms associated with virulence gene expression [9]. Increasingly, nosocomial infections have been linked to *B. thuringiensis* contamination [10]. Illustrating the importance of this microbe as an opportunistic pathogen, some strains have also been observed to be resistant to Ciprofloxacin, a new generation antibiotic used for anthrax treatment and prevention [11].

Within the *B. thuringiensis* genome are genes annotated for simple catechol and complex siderophore production [5]. Genes associated with petrobactin production are found within the *asb* operon, while genes associated with uptake of petrobactin are annotated with the *fat* gene cluster. While much emphasis has focused on the role of petrobactin in physiology and virulence, there are limited studies that specifically address 3,4-dhb and its role in iron acquisition in *Bacillus* species. In some microbes, iron utilization from 3,4-dhb is receptor mediated and has been implicated in virulence [12,13]. This thermostable compound is pH labile with antimicrobial and antifungal activities [14–16]. We hypothesized that 3,4-dhb is produced by *B. thuringiensis* ATCC33679 during growth in iron limited environments. Within the soil, this compound could provide an iron reservoir for growing bacteria. These pockets would also provide the organisms with an area that was limited in competition because of the antimicrobial and antifungal activities.

## 2. Results and Discussion

**Figure 1:**
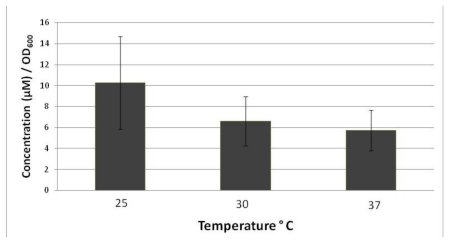
Catechol production is regulated by *B. thuringiensis* growth temperature. Cultures were grown to stationary phase and the optical density of each culture was measured. The Arnow assays were performed on sterile aliquots and the amount of catechol present quantified from a standard curve. Catechol was then normalized by growth (OD_600_). Results indicate the average of four experiments and arrow bars indicate the mean standard deviation. The catechol concentration was 10.25 (±4.42), 6.59 (±2.95) and 5.7 (±1.93) μM/OD_600_ for 25, 30 and 37 °C, respectively.

*B. thuringiensis* was cultured at 25 °C, 30 °C and 37 °C in iron restricted medium, which have proven successful in the generation of siderophore in *Bacillus* species [2,17]. The temperatures were selected to mimic the various environmental conditions that *B. thuringiensis* are routinely isolated from. The lower temperature would represent conditions associated with the soil cycle, while elevated temperatures are associated with virulence factor expression in *B. anthracis* in humans [18]. The intermediate temperature is predicted best for optimal growth, but does not necessarily impact variations in physiology which would associate with enhanced survival or pathogenesis. The amount of iron chelator produced under the various conditions was quantified using the Arnow assay, which is specific for catechols, and normalized to account for any differences in growth [6,17]. Regardless of the growth temperature, iron chelators were detected in culture filtrates. Catechol production was greater at 25 °C (10.25 μM/OD_600_) than at 37 °C (5.7 μM/OD_600_) (Figure 1). Values represent the average of 4 different experiments and the errors represent the variation associated with the final growth yields, which directly impacts the total catechol production and accounts for the broad variations. In *B. anthracis,* catechol production was greater at 37 °C, than at the lower temperature [3]. The difference in the results for *B. thuringiensis versus* those for *B. anthracis* could reflect a difference in species regulation associated with host specificity and environmental adaptation. The differential regulation of genes by *Bacillus* species in response to environmental temperature is well documented [19]. Ecological diversification based on temperature tolerance is predicted to be a key indicator for pathogenicity [20]. In *B. thuringiensis*, lower temperatures are associated with decreased larval survival [21], while 37 °C is the virulence cue for *B. anthracis. B. thuringiensis* multiplication has also been documented in soil, with strains capable of colonizing sterile soil [22]. Thus, iron chelators are important in all of the possible environments encountered by *B. thuringiensis*.

Catechol assays are non-specific, detecting both simple compounds and the more complex siderophores [23]. Typically, both groups are readily observed in iron-deplete environments, with siderophore production occurring later in growth [3]. Both bacillibactin and petrobactin have been isolated from pathogenic and non-pathogenic members of the *B. cereus sensu lato* group [24]. Unlike some microbes, there is significant strain to strain variation within *Bacillus* species for siderophore biosynthesis genes that has not been clearly linked to virulence or ecological adaptations [3]. While *B. thuringiensis* str. 97–27 has putative genes for both siderophores [3], *B. thuringiensis kurstaki* ATCC 33679 lacks *asbB* [25]. Protocatechuic acid production was not explored for this strain and, to our knowledge there have been no discussions of the production of this compound.

**Figure 2:**
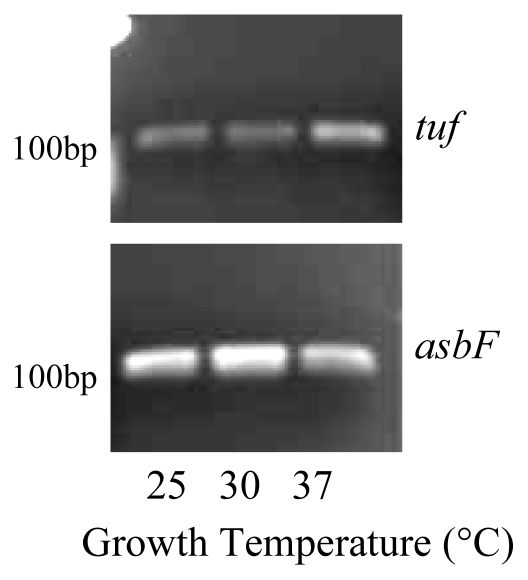
*AsbF* was expressed in *B. thuringiensis* cells grown in iron restricted medium at 25 °C, 30 °C and 37 °C. RNA was isolated from stationary phase cells and reverse transcription PCR with 25 cycles was used to detect gene expression. Experiments were performed in triplicate and a representative gel is shown the internal control *tuf*.

Because protocatechuic acid has been implicated as an important physiological and virulence component for numerous organisms, we speculated that, although petrobactin is not produced, *B. thuringiensis kurstaki* ATCC 33679 does produce the simple catechol protocatechuic acid. While 3,4-dhb was identified by thin layer chromatography in iron restricted culture filtrates (data not shown), we questioned whether the compound was produced as a metabolic byproduct or from the petrobactin operon. *AsbF* is the sixth gene in the petrobactin operon and is responsible for the conversion of (-)-3-dehydroshikimate acid to 3,4-dhb, the iron binding component of petrobactin [6]. Reverse transcription polymerase chain reactions were performed on RNA isolated from whole cells collected using stationary phase growth. The purity and RNA concentration of each sample was measured by Nanodrop and samples were standardized to 5 nanograms for each reaction. A predicted *tuf* reverse transcriptase product was observed at 124 bp in all conditions tested. This elongation factor has been demonstrated to be constitutively expressed in microbial cells and is routinely used as a house keeping gene to monitor mRNA expression levels [26]. The *asbF* product was observed at 115 bp in samples grown under iron-deplete conditions, regardless of the growth temperature (Figure 2). Semi-quantitative RT-PCR was used to identify differences in *asbF* expression under the three growth temperatures. Expression was consistent with Arnow data that indicated that *asbF* expression was lowest at 37 °C.

**Figure 3:**
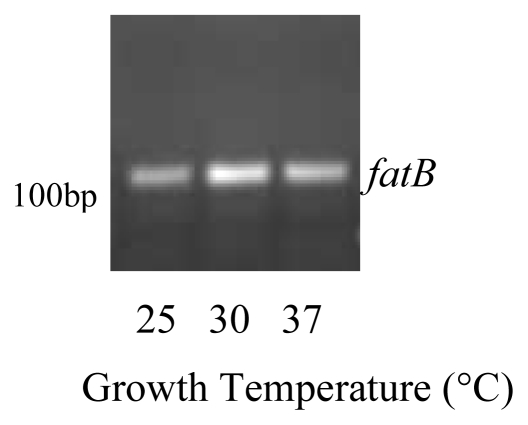
*FatB* was expressed in *B. thuringiensis* cells grown in iron restricted medium at 25 °C, 30 °C and 37 °C. RNA was isolated from stationary phase cells and reverse transcription PCR with 35 cycles was used to detect gene expression. Experiments were performed in triplicate and a representative gel is shown.

Ferri-3,4dhb utilization is typically receptor mediated [12]. Biochemical studies have identified *FatB* as a protein capable of binding 3,4-dhb [27]. *FatB* was expressed in iron restricted *B. thuringiensis* cells grown at the various growth temperatures, although more PCR cycles were required before bands became detectable (Figure 3). The temperature trend observed with *asbF* was not as evident, as expression was actually greater at 30 °C than either 25 °C or 37 °C. It remains unclear how these differences in receptor expression correlate to *asbF*.

## 3. Experimental Section

### 3.1. Microbiological Conditions

*Bacillus thuringiensis kurstaki* ATCC 33679 was purchased from the American Type Culture Collection (ATCC). Cultures were maintained as spores on sporulation agar (23 g nutrient agar, 0.5 g yeast extract, 6.0 mg MnCl_2_, 95.0 mg MgCl_2_, 78.0 mg CaCl_2_ per liter) at 4 °C. For routine growth, bacteria were transferred from the spore stocks to brain-heart infusion (BHIA) slants for overnight incubation at 37 °C. Overnight slants were used to inoculate BHIA slants that were incubated at 37 °C for 5–6 h. These cells were removed from the slants with chelex-treated MM9 (CTM) medium with no added iron [17]. CTM was inoculated at a final concentration of approximately 100 cells per mL and the cultures were incubated with aeration (200 rpm) until stationary phase. Trace metal contamination was decreased on all glassware by rinsing with 6 N hydrochloric acid and deionized water.

### 3.2. Iron Chelator Detection

To detect excretion of iron chelators, aliquots of filtrate were removed from cultures. Samples were centrifuged for 2 min at 14,000 g to remove cells. One mL samples were removed and mixed with 25 μL of ferric chloride (1%). Chelator production is indicated by a color change to either green, purple or red.

### 3.3. Catechol Production

To measure catechol excretion, the Arnow assay was performed on CTM culture filtrates [23]. One mL of filtrate was mixed with 1 mL of 0.5 N HCl, 1 mL of nitrite-molybdate reagent (10 g sodium nitrite, 10 g sodium molybdate per 100 mL) and 1 mL of 1 N NaOH, in this order. The assay was measured spectrophotometrically at A_510_. Catechol production was quantified using an Arnow standard curve of 3,4-dhb (Sigma).

### 3.4. Reverse Transcription Polymerase Chain Reaction (RT-PCR)

To detect 3,4-dhb production, *asbF* expression was monitored under iron restrictive conditions. Total *B. thuringiensis* RNA was isolated from cells in stationary phase according to Fisher SurePrep RNA Purification kit instructions. RNA concentration was measured with the Nanodrop (Thermo Scientific). RNA was used as a template for Invitrogen SuperScript™ One-Step RT-PCR at a concentration of 5 nangrams. The sequence for the forward primer was 5′-CGTGAAACGACAGAA CGAGA-3′ and the reverse primer was 5′-CAGCAAACGTGCGAATTTTA-3′. *Tuf* was also used as a positive control. The forward primer sequence was 5′-GATCACTGGTGCTGCTCAAA-3′ and 5′-TTGCTTCCCAATCAGCTTCT-3′ for the reverse primer. The protocol for cDNA synthesis and amplification are as follows: cDNA synthesis occurred at 50 °C for 25 min, reverse transcriptase enzyme denaturation for 1 min at 94 °C, 30 s for at 94 °C, annealing at 58 °C for 30 s, extension at 68 °C for 1 min. A final extension was done at 72 °C for 5 min after the last cycle. Extension cycles are indicated for each experiment.

## 4. Conclusions

Protocatechuic acid, or 3,4-dhb, is produced under iron restrictive growth conditions in *B. thuringiensis kurstaki* ATCC 33679. Gene expression and catechol release were optimal when cells were grown at ambient temperature. This study marks the first to report the expression of *asbF*, in the absence of *asbB*, which lies in the same operon. This indicates that during the possible soil growth cycle, 3,4-dhb is released into the environmental, sequestering iron and inhibiting microbial growth. *FatB*, the 3,4-dhb receptor, was not regulated in a manner consistent with *asbF*, suggesting that at the different temperatures, the catechol may play different roles. Future studies most focus on elucidating the role between regulation and production of both compounds to elucidate the role of 3,4-dhb in *Bacillus* physiology.
